# Supplementary material for: Contrasting and prioritizing dimensions in ethnic teacher education: A convergent analysis with LDA and fsQCA
Source: PLoS One. 2025 Sep 18;20(9):e0329190. doi: 10.1371/journal.pone.0329190 (PMC12445482; doi:10.1371/journal.pone.0329190)
Supplement: S1 File — (ZIP) [file pone.0329190.s001.zip › PLOS ONE 元数据资料/CiteSpace 结果/narrative_summary.html]

Narrative Summary

# Summary

Time of creation: Tue Apr 15 20:47:38 CST 2025

## Introduction

This summary is automatically generated. To maximize the level of details in the summary, complete the following tasks before running this function:

- clustering and labeling by using the **All in One** button
- run the burst detection function from the **Burstness** tab in the Control Panel
- compute the betweenness centrality from the Nodes menu
- save the current visualization as a PNG image in the project folder

The summary highlights major clusters first, including citing articles and cited references. The importance of nodes will be summarized in terms of citation-based metrics such as citation counts and citation bursts, network-based metrics such as degree centrality and betweenness centrality. Sigma is a combination of both types, i.e., burst and betweenness centrality.

There are other features that are not included in the current summary, for example, structural variation analysis, analysis of uncertainties, concept trees, and dual-map overlays.

## MAJOR CLUSTERS

The network consists of **9** clusters. The largest **9** clusters are summarized as follows.

**Table 1. Summary of the largest 9 clusters.**

| ClusterID | Size | Silhouette | Label (LSI) | Label (LLR) | Label (MI) | Average Year |
| --- | --- | --- | --- | --- | --- | --- |

## CITATION COUNTS

The top ranked item by citation counts is 民族地区 in Cluster #0, with citation counts of **37**. The second one is 教师教育 in Cluster #1, with citation counts of **11**. The third is 少数民族 in Cluster #2, with citation counts of **8**. The 4th is 教师 in Cluster #4, with citation counts of **7**. The 5th is 民族教育 in Cluster #2, with citation counts of **6**. The 6th is 乡村教师 in Cluster #6, with citation counts of **5**. The 7th is 教师队伍 in Cluster #3, with citation counts of **4**. The 8th is 新时代 in Cluster #1, with citation counts of **3**. The 9th is 基础教育 in Cluster #0, with citation counts of **3**. The 10th is 多元文化 in Cluster #9, with citation counts of **3**.

| Citation Counts | Node Name | DOI | Cluster ID |
| --- | --- | --- | --- |
| 37 | 民族地区 |  | 0 |
| 11 | 教师教育 |  | 1 |
| 8 | 少数民族 |  | 2 |
| 7 | 教师 |  | 4 |
| 6 | 民族教育 |  | 2 |
| 5 | 乡村教师 |  | 6 |
| 4 | 教师队伍 |  | 3 |
| 3 | 新时代 |  | 1 |
| 3 | 基础教育 |  | 0 |
| 3 | 多元文化 |  | 9 |

## BURSTS

The top ranked item by bursts is 民族地区 in Cluster #0, with bursts of **0.00**. The second one is 教师教育 in Cluster #1, with bursts of **0.00**. The third is 少数民族 in Cluster #2, with bursts of **0.00**. The 4th is 教师 in Cluster #4, with bursts of **0.00**. The 5th is 民族教育 in Cluster #2, with bursts of **0.00**. The 6th is 乡村教师 in Cluster #6, with bursts of **0.00**. The 7th is 教师队伍 in Cluster #3, with bursts of **0.00**. The 8th is 新时代 in Cluster #1, with bursts of **0.00**. The 9th is 基础教育 in Cluster #0, with bursts of **0.00**. The 10th is 多元文化 in Cluster #9, with bursts of **0.00**.

| Bursts | Node Name | DOI | Cluster ID |
| --- | --- | --- | --- |
| 0.00 | 民族地区 |  | 0 |
| 0.00 | 教师教育 |  | 1 |
| 0.00 | 少数民族 |  | 2 |
| 0.00 | 教师 |  | 4 |
| 0.00 | 民族教育 |  | 2 |
| 0.00 | 乡村教师 |  | 6 |
| 0.00 | 教师队伍 |  | 3 |
| 0.00 | 新时代 |  | 1 |
| 0.00 | 基础教育 |  | 0 |
| 0.00 | 多元文化 |  | 9 |

## DEGREE

The top ranked item by degree is 民族地区 in Cluster #0, with degree of **93**. The second one is 教师教育 in Cluster #1, with degree of **28**. The third is 少数民族 in Cluster #2, with degree of **23**. The 4th is 民族教育 in Cluster #2, with degree of **15**. The 5th is 教师队伍 in Cluster #3, with degree of **14**. The 6th is 教师 in Cluster #4, with degree of **13**. The 7th is 数学教育 in Cluster #5, with degree of **10**. The 8th is 乡村教师 in Cluster #6, with degree of **9**. The 9th is 新时代 in Cluster #1, with degree of **9**. The 10th is 中央民族大学教育学院 in Cluster #8, with degree of **9**.

| Degree | Node Name | DOI | Cluster ID |
| --- | --- | --- | --- |
| 93 | 民族地区 |  | 0 |
| 28 | 教师教育 |  | 1 |
| 23 | 少数民族 |  | 2 |
| 15 | 民族教育 |  | 2 |
| 14 | 教师队伍 |  | 3 |
| 13 | 教师 |  | 4 |
| 10 | 数学教育 |  | 5 |
| 9 | 乡村教师 |  | 6 |
| 9 | 新时代 |  | 1 |
| 9 | 中央民族大学教育学院 |  | 8 |

## CENTRALITY

The top ranked item by centrality is 民族地区 in Cluster #0, with centrality of **0.78**. The second one is 教师教育 in Cluster #1, with centrality of **0.17**. The third is 教师队伍 in Cluster #3, with centrality of **0.16**. The 4th is 少数民族 in Cluster #2, with centrality of **0.10**. The 5th is 教师 in Cluster #4, with centrality of **0.10**. The 6th is 民族教育 in Cluster #2, with centrality of **0.08**. The 7th is 双语教育 in Cluster #3, with centrality of **0.05**. The 8th is 新时代 in Cluster #1, with centrality of **0.04**. The 9th is 北京师范大学教育学部 in Cluster #2, with centrality of **0.04**. The 10th is 乡村教师 in Cluster #6, with centrality of **0.03**.

| Centrality | Node Name | DOI | Cluster ID |
| --- | --- | --- | --- |
| 0.78 | 民族地区 |  | 0 |
| 0.17 | 教师教育 |  | 1 |
| 0.16 | 教师队伍 |  | 3 |
| 0.10 | 少数民族 |  | 2 |
| 0.10 | 教师 |  | 4 |
| 0.08 | 民族教育 |  | 2 |
| 0.05 | 双语教育 |  | 3 |
| 0.04 | 新时代 |  | 1 |
| 0.04 | 北京师范大学教育学部 |  | 2 |
| 0.03 | 乡村教师 |  | 6 |

## SIGMA

The top ranked item by sigma is 民族地区 in Cluster #0, with sigma of **1.00**. The second one is 教师教育 in Cluster #1, with sigma of **1.00**. The third is 教师队伍 in Cluster #3, with sigma of **1.00**. The 4th is 少数民族 in Cluster #2, with sigma of **1.00**. The 5th is 教师 in Cluster #4, with sigma of **1.00**. The 6th is 民族教育 in Cluster #2, with sigma of **1.00**. The 7th is 双语教育 in Cluster #3, with sigma of **1.00**. The 8th is 新时代 in Cluster #1, with sigma of **1.00**. The 9th is 北京师范大学教育学部 in Cluster #2, with sigma of **1.00**. The 10th is 乡村教师 in Cluster #6, with sigma of **1.00**.

| Sigma | Node Name | DOI | Cluster ID |
| --- | --- | --- | --- |
| 1.00 | 民族地区 |  | 0 |
| 1.00 | 教师教育 |  | 1 |
| 1.00 | 教师队伍 |  | 3 |
| 1.00 | 少数民族 |  | 2 |
| 1.00 | 教师 |  | 4 |
| 1.00 | 民族教育 |  | 2 |
| 1.00 | 双语教育 |  | 3 |
| 1.00 | 新时代 |  | 1 |
| 1.00 | 北京师范大学教育学部 |  | 2 |
| 1.00 | 乡村教师 |  | 6 |

## References

- Chen, C. (2004) Searching for intellectual turning points: Progressive knowledge domain visualization. PNAS, 101 (suppl\_1), 5303-5310. 10.1073/pnas.0307513100 **CiteSpace Original**
- Chen, C. (2006) CiteSpace II: Detecting and visualizing emerging trends and transient patterns in scientific literature. Journal of the American Society for Information Science and Technology, 57(3), 359-377. 10.1002/asi.20317 **CiteSpace II**
- Chen, C., Ibekwe-SanJuan, F., Hou, J. (2010) The structure and dynamics of cocitation clusters: A multiple‐perspective cocitation analysis. Journal of the American Society for information Science and Technology, 61(7), 1386-1409. 10.1002.asi.21309 **cluster labeling**
- Chen, C. (2012) Predictive effects of structural variation on citation counts. Journal of the American Society for Information Science and Technology, 63(3), 431-449. 10.1002/asi.21694 **structural variation analysis**
- Chen, C. (2017) Science Mapping: A Systematic Review of the Literature. Journal of Data and Information Science, 2(2), 1-40. 10.1515/jdis-2017-0006 **a show-case study**
- Chen, C., Song, M. (2019) Visualizing a Field of Research: A Methodology of Systematic Scientometric Reviews. PLoS One, 14(10), e0223994. 10.1371/journal.pone.0223994 **cascading citation expansion**
- Chen, C. (2020) A Glimpse of the First Eight Months of the COVID-19 Literature on Microsoft Academic Graph: Themes, Citation Contexts, and Uncertainties. Frontiers in Research Metrics and Analytics, 5:607286. 10.3389/frma.2020.607286 **citation contexts; uncertainties**
