# Supplementary material for: Contrasting and prioritizing dimensions in ethnic teacher education: A convergent analysis with LDA and fsQCA
Source: PLoS One. 2025 Sep 18;20(9):e0329190. doi: 10.1371/journal.pone.0329190 (PMC12445482; doi:10.1371/journal.pone.0329190)
Supplement: S1 File — (ZIP) [file pone.0329190.s001.zip › PLOS ONE 元数据资料/CiteSpace 结果/ORG_cluster_summary.html]

Cluster Summary (9 Clusters by Size)

|  |  |  |  |  |  |  |  |
| --- | --- | --- | --- | --- | --- | --- | --- |
| **Selected** | **ClusterID** | **Size** | **Silhouette** | **mean(Year)** | **Label (LSI)** | **Label (LLR)** | **Label (MI)** |
| false | 理科课程 (1.18); 现状 (1.18); 中小学教师 (1.18); 藏族中学生 (1.18); 实践取向 (1.18) | (11.17) 中小学; (8.64) 在线教学; | 在线教学 (3.93, 0.05); 中小学 (3.93, 0.05); 基础教育 (3.93, 0.05); 理科教育 (3.93, 0.05); 教育信息化 (3.93, 0.05) | 0 | 40 | 0.978 | 2020 |
| true | 民国时期 (0.26); 教师专业发展 (0.26); 边疆民族地区 (0.26); 区域教育 (0.26); 城乡数字资源不均衡 (0.26) | (12.97) 教师教育; | 教师教育 (17.34, 1.0E-4); 民国时期 (4.15, 0.05); 教师专业发展 (4.15, 0.05); 边疆民族地区 (4.15, 0.05); 区域教育 (4.15, 0.05) | 1 | 35 | 0.913 | 2020 |
| false | 民族地区 (0.07); 卓越教师 (0.06); 个案 (0.06); 叙事 (0.06); 教育信息技术 (0.06) | (4.32) 卓越教师; | 卓越教师 (6.63, 0.05); 个案 (6.63, 0.05); 叙事 (6.63, 0.05); 教育信息技术 (6.63, 0.05); 教师教育 (0.33, 1.0) | 8 | 8 | 0.94 | 2015 |
| false | 幼儿 (0.07); 政策建议 (0.07); 教育质量 (0.07); 普通话能力 (0.07); 城乡差异 (0.07) | (4.32) 政策建议; | 幼儿 (6.22, 0.05); 政策建议 (6.22, 0.05); 教育质量 (6.22, 0.05); 普通话能力 (6.22, 0.05); 城乡差异 (6.22, 0.05) | 7 | 9 | 0.98 | 2023 |
| false | 数学学业成绩 (0.07); 课堂教学质量 (0.07); 多层线性模型 (0.07); 数学教育 (0.07); mpck (0.07) | (4.32) 数学学业成绩; | 数学学业成绩 (6.22, 0.05); 课堂教学质量 (6.22, 0.05); 多层线性模型 (6.22, 0.05); 数学教育 (6.22, 0.05); mpck (6.22, 0.05) | 5 | 10 | 0.975 | 2023 |
| false | 铸牢中华民族共同体意识 (0.07); 教师 (0.07); 师资培训 (0.07); 民族幼儿园 (0.07); 教师编制 (0.07) | (4.32) 铸牢中华民族共同体意识; | 铸牢中华民族共同体意识 (6.22, 0.05); 教师 (6.22, 0.05); 师资培训 (6.22, 0.05); 民族幼儿园 (6.22, 0.05); 教师编制 (6.22, 0.05) | 4 | 14 | 0.948 | 2017 |
| false | 学科专业建设 (0.22); 协同育人 (0.22); 民族和睦 (0.22); 教学改革 (0.22); 教师队伍 (0.22) | (8.21) 培养; | 民族地区 (4.97, 0.05); 学科专业建设 (4.43, 0.05); 协同育人 (4.43, 0.05); 民族和睦 (4.43, 0.05); 教学改革 (4.43, 0.05) | 3 | 17 | 0.997 | 2018 |
| false | 体系 (0.09); 终极责任 (0.09); 培养模式 (0.09); 教育信念 (0.09); 乡村教师 (0.08) | (8.64) 乡村教师; (4.32) 体系; (4.32) 终极责任 | 乡村教师 (12.1, 0.001); 体系 (5.89, 0.05); 终极责任 (5.89, 0.05); 培养模式 (5.89, 0.05); 教育信念 (5.89, 0.05) | 6 | 9 | 0.985 | 2020 |
| false | 双语教师 (0.28); 教育素养 (0.28); 理科学习 (0.28); 强师计划 (0.28); 语言和文化适宜 (0.28) | (8.64) 少数民族; (4.32) 双语教师; | 培养 (8.16, 0.005); 少数民族 (8.16, 0.005); 双语教师 (4.02, 0.05); 教育素养 (4.02, 0.05); 理科学习 (4.02, 0.05) | 2 | 29 | 0.94 | 2017 |
