# Supplementary figures and images for: Contrasting and prioritizing dimensions in ethnic teacher education: A convergent analysis with LDA and fsQCA
Source: PLoS One. 2025 Sep 18;20(9):e0329190. doi: 10.1371/journal.pone.0329190 (PMC12445482; doi:10.1371/journal.pone.0329190)

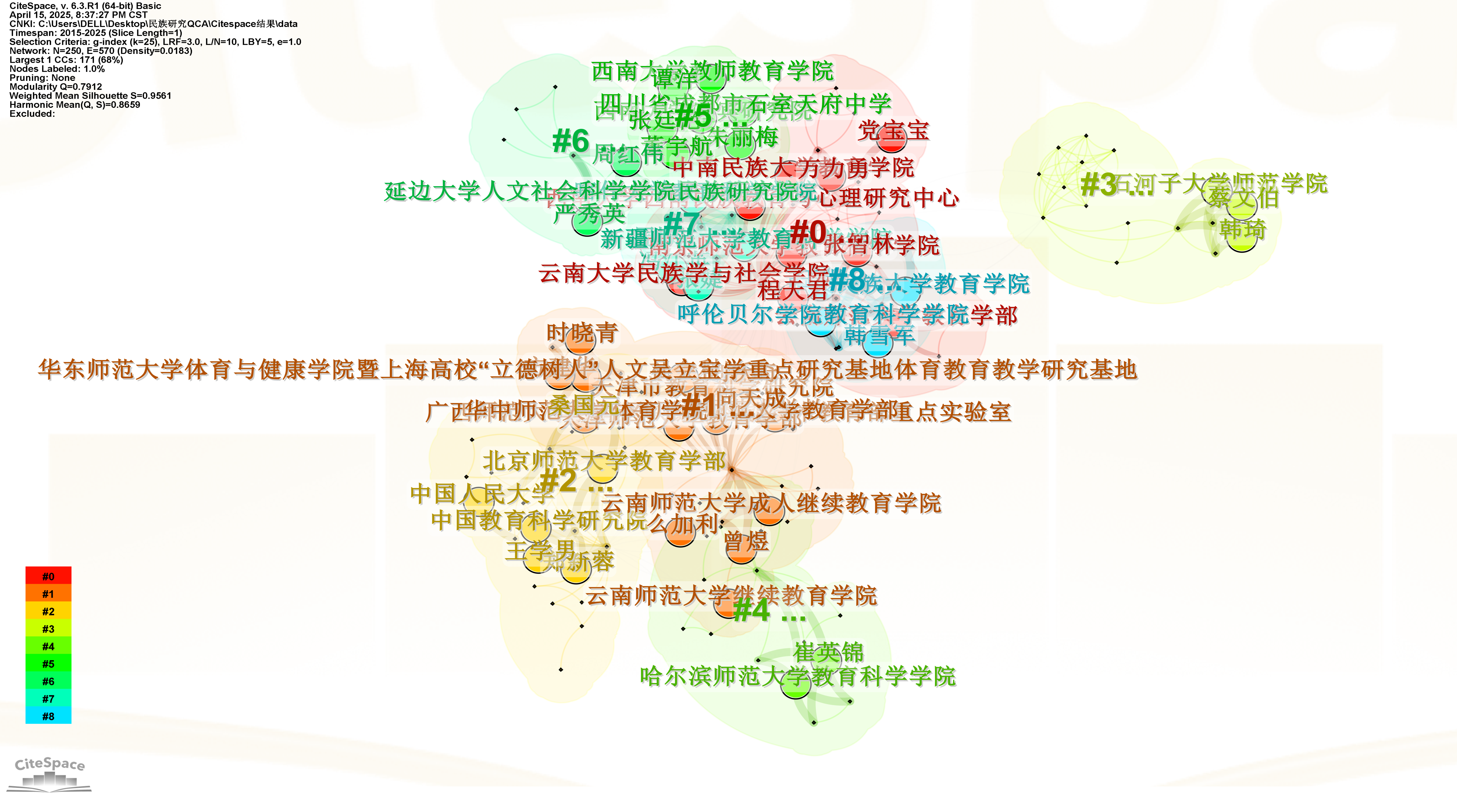

Supplement: S1 File — (ZIP) [file pone.0329190.s001.zip › PLOS ONE 元数据资料/CiteSpace 结果/ORG_v250e570.png]
