# Supplementary material for: Contrasting and prioritizing dimensions in ethnic teacher education: A convergent analysis with LDA and fsQCA
Source: PLoS One. 2025 Sep 18;20(9):e0329190. doi: 10.1371/journal.pone.0329190 (PMC12445482; doi:10.1371/journal.pone.0329190)
Supplement: Attachment — Submitted filename: REview Artikel Plose One.docx [file pone.0329190.s002.docx]

**Reviewer Recommendation and Comments for Manuscript Number PONE-D-25-25749**

9 Juni 2025

**Contrasting and Prioritizing Dimensions in Ethnic Teacher Education: A Convergent Analysis with LDA and fsQCA**

I. **ABSTRACT ANALYSIS**

Abstract was already Systematic and complete. Has a background, specific research objectives, and appropriate methodology

**Abstract Improvement Suggestions:**

1. Add data sources and sampling techniques concisely: “...based on a sample of 250 peer-reviewed articles retrieved from the CNKI database…”
2. Emphasize the purpose in the opening sentence: Add one sentence stating the gap in previous research and the unique contribution of this study. Example: “Previous studies have rarely examined ethnic teacher education from a configurational perspective. This study addresses that gap…”
3. Emphasize the unique novelty this research at the end of abstract
4. Clarify theoretical and practical contributions more explicitly: Use sentences such as: “The findings contribute to both theory and policy by proposing a novel framework for…”
5. Language can be condensed to be more focused: Some sentences in the middle of the abstract are rather long and can be made more concise without reducing the meaning.
II. **INTRODUCTION ANALYSIS**

**Strengths:**

1. Systematic (Explanation starts from the importance of ethnic education → role of teachers → government policy → challenges in the field).
2. Logical writing (The sequence flows logically: from policy → challenges → research gaps → new approaches).
3. Relevant and up-to-date topics (Bringing up policies from 2018 and 2022. This is relevant and current in the Chinese context).
4. There is a clear research gap: It is stated that previous studies: are too retrospective, only assess current practices, do not address future needs, and are not systemic.
5. References: Some references are up-to-date (2018–2022), but some are too old or general (e.g. Cremin's theory from the 1970s). Some also come from local journals or Chinese sources, not international journals.

**Weaknesses**

1. The formulation of the problem is not explicit.

The article explains the challenges and objectives, but does not formulate the research question in an explicit sentence such as: "This study aims to answer the question: how does the configuration of variables... affect the effectiveness of..."

2. Lack of reinforcement from international literature.

The explanation uses a lot of data and studies from China. It would be stronger if there was a comparison with similar international contexts (e.g., teacher education in indigenous communities, Africa, Latin America).

**Suggestions for Improvement**

1. Add an explicit statement of the problem statement: For example: “Given the fragmented understanding of ethnic teacher education in China, this study addresses the following research questions: (1) What are the core thematic dimensions of ethnic teacher education? (2) How do combinations of these dimensions affect the effectiveness of ethnic teacher education?”

2. Strengthen the international literature base: Add 1–2 references from international contexts (e.g., from the International Journal of Educational Development or Comparative Education) to demonstrate global relevance.

3. Rationalize the approach more explicitly: Explain why the combination of LDA + fsQCA is more appropriate than classical quantitative approaches (e.g., regression), especially for the complexity of ethnic regions.

**III. METHODOLOGY ANALYSIS**

**Strength**

1. Innovative approach and fit with the research question: fsQCA is very appropriate for looking at combinations of variables in complex contexts such as ethnic teacher education. LDA helps to find hidden themes in a data-driven way, without researcher bias.

2. Utilization of big data: Using 250 articles over 10 years provides a strong basis for identifying trends.

3. Validation of results is done through robust tests (increasing the PRI threshold in fsQCA).

**Weaknesses**

1. There is no validation process for the quality of articles used as data sources. It is not stated whether the articles went through blind review, categorization of articles from locally indexed journals, or came from, Scopus, etc.

2. Subjectivity in fuzzy-set calibration. Although using a reference (0.75 / 0.5 / 0.25), the mapping of values from articles to the scale is still manual and can be biased.

3. Does not include triangulation or inter-coder validation. There is no inter-rater reliability check if there are several people reading and assessing the article.

4. No mention of software testing or training (ROST/CiteSpace/LDA) for model validation or parameter settings.

**Suggestions for Methodology Improvement**

1. Add article inclusion-exclusion criteria: For example: “Only peer-reviewed journal articles related to ethnic teacher training in mainland China were included…”

2. Use supporting instruments such as checklists: For example: coding tools to assess the relevance, methodological quality, and focus of the selected articles.

3. Explain the data selection validation process: Who selected? Was double-blind screening done? Were there other reviewers?

4. Add references to the quality of the LDA and fsQCA methods: So that it can be understood why the K5 parameters were selected and how the fsQCA was calibrated.

5. Expand data triangulation: Add, for example, interviews with ethnic teacher lecturers or policy documents, so that the findings are stronger and not only based on academic literature.

**IV. DISCUSSION ANALYSIS**

**Strengths and weaknesses**

1. The discussion is quite complete: 1) Explains the results of fsQCA in detail, including: Six configurations (3 high effective, 3 low effective), Determinants and their relationships with each other; 2) Accompanied by interpretation of each configuration, both success and failure conditions; 3) Theoretical and practical implications are provided.

However, there are some incomplete ones: • Does not discuss differences between ethnic regions specifically (eg Tibet vs Xinjiang vs Guizhou); • Does not explore the possible role of educational actors such as principals, communities, or NGOs;

2. Has strong statistical data: • Uses data from 250 scientific articles filtered from CNKI; • LDA analysis produces 5 dominant topics based on the perplexity and coherence models; • fsQCA uses fuzzy-set scoring based on word frequency and correlation; • Coverage, consistency values, and a very complete table of result configurations are available.

However: • There is no statistical data in quantitative field form (e.g. teacher survey results, questionnaires, or hypothesis tests). • All analyses are based on secondary literature — this makes the data technically strong, but less empirically direct.

3. Some results are linked to appropriate references: • The discussion is quite deep in terms of internal interpretation of the data. • However, it is rare to explicitly link the findings to previous studies, especially: Not comparing whether the results are similar or different from other studies. There are no citations explaining the position of this article among similar articles (both national and international).

4. The references are sufficient in number, but not optimal in terms of supporting the results. • There are around 15 references, mostly local literature (China). • There are almost no international references or meta-analyses of ethnic education. • Not used to strengthen the results, more as a general background or methodology.

**Suggestions for Improvement of Discussion**

1. Strengthen connections with relevant literature, especially: 1) Add comparisons with studies from other countries (eg: teacher education for indigenous communities in Canada, indigenous tribes in Australia, etc.); 2) Use previous study citations to support or contrast the results of the fsQCA configuration.

2. Include theoretical references when explaining the configuration of results, For example, when mentioning the importance of teacher development and cultural education - connect it with the theory of culturally responsive pedagogy (Gay, 2010) or teacher agency.

3. Provide additional contextual data, such as: 1) Demographic conditions of ethnic areas, 2) Challenges of teacher distribution, 3) Training gaps between regions - so that readers get a real picture.

4. Add citations from national reports or surveys (if any) that describe the situation of ethnic teachers in the field.

5. Provide an explicit statement about the limitations of generalizing the results from secondary literature.

**V. CONCLUSION ANALYSIS**

Although the RQ is not explicitly written, but the conclusion answers clearly: thematic dimensions are important + how the combination of factors works in shaping educational effectiveness. Novelty is seen through the combination method (LDA + fsQCA) and the configuration of the results. However, it does not explicitly state the scientific contribution that distinguishes this study from others. Already mentioning the shortcomings of the study (1) data only from secondary literature, (2) fsQCA calibration is subjective. Also provides suggestions for further research (using mixed methods: field surveys, automated NLP to reduce human bias, ethnographic studies in the field). The writing is solid, organized, and concise - making it easier for readers to understand the direction of the research and its applications

**Suggestions for Improving the Conclusion**

1. Add an explicit sentence about novelty:

2. State the theoretical contribution explicitly: For example: expanding the understanding of the teaching behavior-learning outcome model in the context of multicultural education.

3. Emphasize the position of the results in the academic landscape: Do these results support or challenge previous findings?

4. Provide a follow-up research question: Example: "Future research should explore how local cultural identity directly affects the professional agency of ethnic teachers using field-based ethnographic methods."
