# Supplementary material for: Contrasting and prioritizing dimensions in ethnic teacher education: A convergent analysis with LDA and fsQCA
Source: PLoS One. 2025 Sep 18;20(9):e0329190. doi: 10.1371/journal.pone.0329190 (PMC12445482; doi:10.1371/journal.pone.0329190)
Supplement: Attachment — Submitted filename: Respond to Reviewers.docx [file pone.0329190.s004.docx]

# Respond to Reviewers

## 1. **ABSTRACT**

1.*Add data sources and sampling techniques concisely: “...based on a sample of 250 peer-reviewed articles retrieved from the CNKI database…”*

A: We have added the following to the abstract: 'This study analyzes 250 peer-reviewed articles from PKU/CSSCI-indexed journals in the CNKI database (2015–2025), identified using keywords such as "ethnic teacher education," "ethnic education," and "ethnic teachers." These were refined from an initial pool of 2,672 records through manual screenin.

2.*Emphasize the purpose in the opening sentence: Add one sentence stating the gap in previous research and the unique contribution of this study. Example: “Previous studies have rarely examined ethnic teacher education from a configurational perspective. This study addresses that gap…”*

A: We have added the following to the abstract: “Previous studies have rarely examined ethnic teacher education from a configurational perspective. This study addresses that gap.”

3*.Emphasize the unique novelty this research at the end of abstract*

A: We have added the following to the abstract: “This study introduces the novel integration of LDA topic modeling and fsQCA in educational research. While LDA uncovers latent themes and fsQCA examines causal complexity, their combined application enables simultaneous discovery and validation of configurations a previously unexplored approach in ethnic teacher education.”

4.*Clarify theoretical and practical contributions more explicitly: Use sentences such as: “The findings contribute to both theory and policy by proposing a novel framework for…”*

A: We have added the following to the abstract: “These findings make dual contributions: theoretically, by advancing a novel conceptual framework; and practically, by yielding actionable policy implications for ethnic teacher education development.”

5.*Language can be condensed to be more focused: Some sentences in the middle of the abstract are rather long and can be made more concise without reducing the meaning.*

A: We have restructured the abstract, converting lengthy single sentences into concise, digestible statements.

## 1. INTRODUCTION

1. *Add an explicit statement of the problem statement: For example: “Given the fragmented understanding of ethnic teacher education in China, this study addresses the following research questions: (1) What are the core thematic dimensions of ethnic teacher education? (2) How do combinations of these dimensions affect the effectiveness of ethnic teacher education?”*

A: We have added the following to the introduction “Given the fragmented understanding of ethnic teacher education in China, this study examines: (1) the core thematic dimensions of ethnic teacher education, and (2) how their configurations influence educational effectiveness.

2. *Strengthen the international literature base: Add 1–2 references from international contexts (e.g., from the International Journal of Educational Development or Comparative Education) to demonstrate global relevance.*

A: The references section has been reorganized and expanded to include more internationally contextualized sources.

3. *Rationalize the approach more explicitly: Explain why the combination of LDA + fsQCA is more appropriate than classical quantitative approaches (e.g., regression), especially for the complexity of ethnic regions.*

A: We have added the following to the introduction “ This study innovatively integrates LDA and fsQCA to overcome regression analysis' limitations in studying ethnic teacher education. Unlike regression's linear assumptions, our approach captures system complexity and equifinality through two phases: LDA extracts cultural themes from qualitative data, then fsQCA analyzes their configurations. Key advantages include: (1) small-N robustness with contextual sensitivity , (2) identification of causal asymmetries, and (3) multiple success pathways for diverse contexts. This advances understanding of ethnic education's complex causality beyond variable-centered methods.”

## **3. METHODOLOGY**

*1. Add article inclusion-exclusion criteria: For example: “Only peer-reviewed journal art6b icles related to ethnic teacher training in mainland China were included…”*

A: The exclusion criteria for article selection have been elaborated with greater specificity.“This study exclusively analyzed peer-reviewed journal articles focusing on teacher training programs within Mainland China. ”

*2.Use supporting instruments such as checklists: For example: coding tools to assess the relevance, methodological quality, and focus of the selected articles.*

A: We employed a simple scale tool as a reference during the screening process.We have added the following to the methodology “ Article selection followed PRISMA guidelines for duplicate removal, with two researchers independently screening studies using the CASP Qualitative Checklist. Discrepancies were resolved through consensus.”

4. *Explain the data selection validation process: Who selected? Was double-blind screening done? Were there other reviewers?*

A: We have added the following to the methodology “ Article selection followed PRISMA guidelines for duplicate removal, with two researchers independently screening studies using the CASP Qualitative Checklist. Discrepancies were resolved through consensus.”

5. *Add references to the quality of the LDA and fsQCA methods: So that it can be understood why the K5 parameters were selected and how the fsQCA was calibrated.*

A: We have provided more detailed citations and explanations regarding the perplexity and consistency curves on pages 11-12 of the main text.

6. *Expand data triangulation: Add, for example, interviews with ethnic teacher lecturers or policy documents, so that the findings are stronger and not only based on academic literature.*

A: We sincerely appreciate your insightful suggestions and fully acknowledge their validity. However, due to current limitations in our geographic location, research funding, and personnel availability, we are unable to conduct interviews at this stage. We have therefore explicitly addressed this constraint in the Discussion section and identified it as an important direction for future research.

## 4. DISCUSSION

*1. Strengthen connections with relevant literature, especially: 1) Add comparisons with studies from other countries (eg: teacher education for indigenous communities in Canada, indigenous tribes in Australia, etc.); 2) Use previous study citations to support or contrast the results of the fsQCA configuration.*

A: We have incorporated the following additions to strengthen the international and methodological dimensions of our study: Page 25: Added a case study on Indigenous education in Canada;Page 26: Included comparative analysis with fsQCA (Fuzzy-Set Qualitative Comparative Analysis) computational results

*2. Include theoretical references when explaining the configuration of results, For example, when mentioning the importance of teacher development and cultural education - connect it with the theory of culturally responsive pedagogy (Gay, 2010) or teacher agency.*

A: We have conducted a linkage analysis of culturally responsive pedagogy on page 22 of the main text, with supporting citations (Citation 29).

*3. Provide additional contextual data, such as: 1) Demographic conditions of ethnic areas, 2) Challenges of teacher distribution, 3) Training gaps between regions - so that readers get a real picture.*

A: We have included an analysis of educational realities and research disparities in China's ethnic regions on page 22 of the manuscript.

*4. Add citations from national reports or surveys (if any) that describe the situation of ethnic teachers in the field.*

A: We sincerely appreciate your constructive suggestions. After carefully reviewing numerous national reports and academic publications, we were unable to identify suitable references that directly align with the proposed modifications. While we fully acknowledge the value of your recommendations, current limitations in available literature prevent their immediate implementation. We will prioritize this as a key objective for our ongoing research program.

5. *Provide an explicit statement about the limitations of generalizing the results from secondary literature.*

A: We have addressed the limitations of secondary literature research on page 26 of the manuscript and outlined plans to enhance data diversity in future studies.

## 5.CONCLUSION

*1. Add an explicit sentence about novelty:*

A: We have articulated the study's novel contributions in a dedicated section on page 25 of the manuscript.This study pioneers an innovative methodological integration of Latent Dirichlet Allocation (LDA) topic modeling and fuzzy-set Qualitative Comparative Analysis (fsQCA) in educational research. While LDA serves to extract latent thematic dimensions from textual data, fsQCA systematically analyzes how these dimensions combine to produce causal outcomes. Their synergistic application enables both the discovery of emergent patterns and the validation of configurational hypotheses representing a significant methodological advance in ethnic teacher education research.

*2. State the theoretical contribution explicitly: For example: expanding the understanding of the teaching behavior-learning outcome model in the context of multicultural education.*

A: We have elaborated on the study's theoretical contributions across pages 25-26 of the manuscript.

*3. Emphasize the position of the results in the academic landscape: Do these results support or challenge previous findings?*

A: We have addressed the study's academic positioning through a comparative analysis on page 23 of the manuscript.

*4. Provide a follow-up research question: Example: "Future research should explore how local cultural identity directly affects the professional agency of ethnic teachers using field-based ethnographic methods."*

A:We have dedicated the final section of the manuscript to outlining future research directions, with your valuable suggestions prioritized as key investigative pathways. Future research should adopt a multi-dimensional approach to investigate the complex interplay between local cultural identity and ethnic teachers' professional agency. Specifically, longitudinal ethnographic studies could: Trace how teachers navigate between indigenous knowledge systems (e.g., oral traditions, community values) and standardized pedagogical requirements; Document agentic practices through participant observation in both formal (classrooms) and informal (community gatherings) settings.
